# Quercetin Suppresses Apoptosis and Attenuates Intervertebral Disc Degeneration via the SIRT1-Autophagy Pathway

**DOI:** 10.3389/fcell.2020.613006

**Published:** 2020-12-10

**Authors:** Dong Wang, Xin He, Di Wang, Pandi Peng, Xiaolong Xu, Bo Gao, Chao Zheng, Han Wang, Haoruo Jia, Qiliang Shang, Zhen Sun, Zhuojing Luo, Liu Yang

**Affiliations:** ^1^Institute of Orthopedic Surgery, Xijing Hospital, Fourth Military Medical University, Xi’an, China; ^2^Department of Medicine Chemistry and Pharmaceutical Analysis, School of Pharmacy, Fourth Military Medical University, Xi’an, China; ^3^Medical Research Institute, Northwestern Polytechnical University, Xi’an, China

**Keywords:** quercetin, oxidative stress, intervertebral disc degeneration, autophagy, SIRT1

## Abstract

Intervertebral disc degeneration (IDD) has been generally accepted as the major cause of low back pain (LBP), which causes an enormous socioeconomic burden. Previous studies demonstrated that the apoptosis of nucleus pulposus (NP) cells and the dyshomeostasis of extracellular matrix (ECM) contributed to the pathogenesis of IDD, and effective therapies were still lacking. Quercetin, a natural flavonoid possessing a specific effect of autophagy stimulation and SIRT1 activation, showed some protective effect on a series of degenerative diseases. Based on previous studies, we hypothesized that quercetin might have therapeutic effects on IDD by inhibiting the apoptosis of NP cells and dyshomeostasis of ECM via the SIRT1-autophagy pathway. In this study, we revealed that quercetin treatment inhibited the apoptosis of NP cells and ECM degeneration induced by oxidative stress. We also found that quercetin promoted the expression of SIRT1 and autophagy in NP cells in a dose-dependent manner. Autophagy inhibitor 3-methyladenine (3-MA) reversed the protective effect of quercetin on apoptosis and ECM degeneration. Moreover, SIRT1 enzymatic activity inhibitor EX-527, suppressed quercetin-induced autophagy and the protective effect on NP cells, indicating that quercetin protected NP cells against apoptosis and prevented ECM degeneration via SIRT1-autophagy pathway. *In vivo*, quercetin was also demonstrated to alleviate the progression of IDD in rats. Taken together, our results suggest that quercetin prevents IDD by promoting SIRT1-dependent autophagy, indicating one novel and effective therapeutic method for IDD.

## Introduction

Low back pain (LBP) is one of the most common musculoskeletal diseases that leads to a low quality of life and high socioeconomic burden (Disease et al., 2016; Global Burden of Disease Cancer, Fitzmaurice et al., 2017; Hurwitz et al., 2018). Intervertebral disc degeneration (IDD) has been generally accepted as the major cause of LBP (Sun et al., 2013; Molladavoodi et al., 2019; Taylor and Bishop, 2019). However, there are few efficacious drugs to prevent the occurrence and development of IDD.

Intervertebral discs (IVDs) are composed of the gelatinous inner nucleus pulposus (NP), the external annulus fibrosus (AF) and the upper and lower cartilage endplates (Gopal et al., 2012; Kepler et al., 2013; Vergroesen et al., 2015). NP cells, the major population in NP tissues, play a critical role in maintaining homeostasis of extracellular matrix (ECM), which contributes to confront diverse mechanical loading and deformation (Bian et al., 2017; Zhang et al., 2018b; Gao et al., 2020). Some previous studies suggested that NP dysfunction was closely related to IDD pathogenesis (Cheng et al., 2018; Ji et al., 2018). Some extracellular stimuli such as hypoxia, nutritional deprivation, inflammation or mechanical loading, could trigger the apoptosis and an imbalance between anabolic and catabolic activities of NP cell, and then primed the process of IDD (Ma et al., 2013; Choi et al., 2016; Zhang et al., 2018b, 2019; Yurube et al., 2019; Zuo et al., 2019).

Oxidative stress is one major cause for many degenerative diseases (Tang et al., 2017; Sena et al., 2018; Zhang et al., 2018a). Some studies have indicated oxidative stress can induce apoptosis of NP cells and degradation of ECM, which play significant role in the pathogenesis of IDD (Chu et al., 2016; Chen et al., 2017; Tang et al., 2019). Therefore, inhibition of oxidative stress-induced apoptosis of NP cells and degradation of ECM might be a potential therapeutic target for IDD.

Autophagy is an evolutionarily conserved cellular behavior through which the aberrant organelles and proteins induced by cellular stress can be eliminated (Mizushima and Komatsu, 2011; Wang et al., 2019). Previous studies showed that autophagy played a protective role for IDD, and activation of autophagy might be an efficacious treatment (Ye et al., 2018; Kang et al., 2019). Recent studies reported that the expression and activation of SIRT1, a NAD^+^-dependent deacetylase, might play an important protective role in maintaining IVDs homeostasis (Jiang et al., 2014; Wang et al., 2016; Zhang et al., 2019). Moreover, SIRT1 also showed an ability to activate autophagy in some other tissues (Hariharan et al., 2010; Kume et al., 2010; Wang et al., 2012).

**FIGURE 1:**
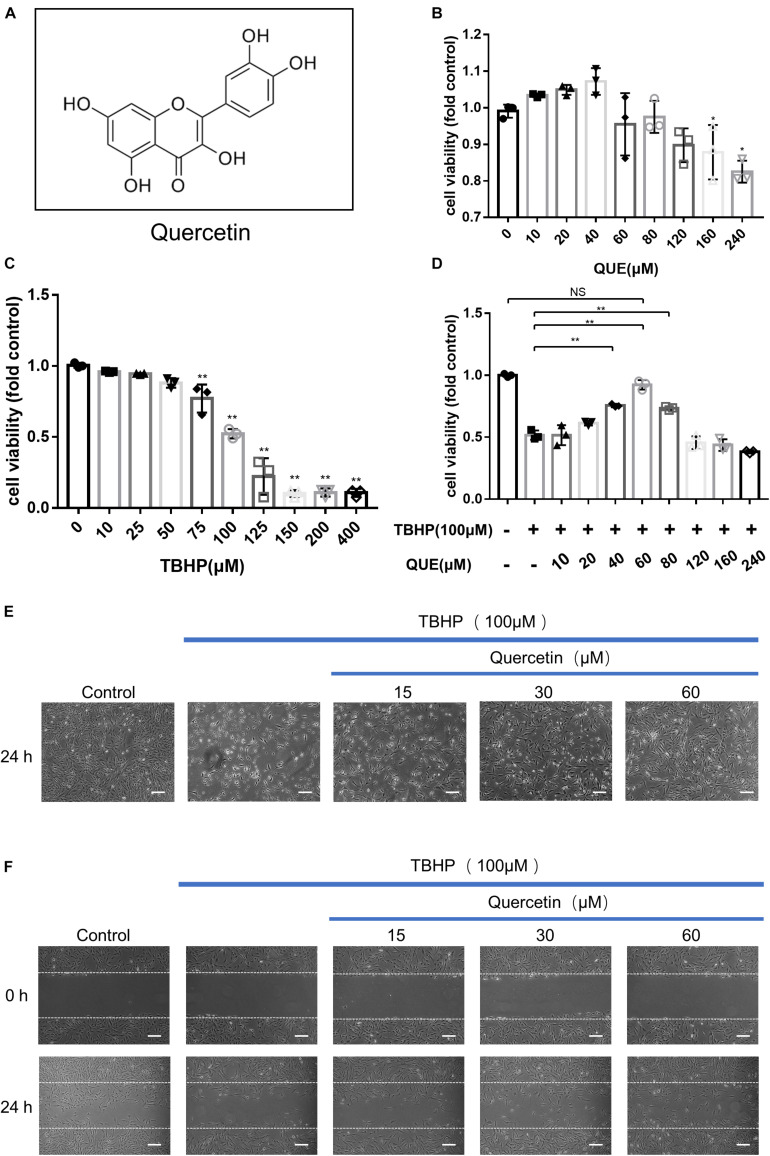
Effects of quercetin and TBHP on NP cells viability. **(A)** Chemical structure of quercetin; **(B,C)** Cell Counting Kit-8 (CCK-8) results of the NP cells viability with different concentrations of quercetin and TBHP for 24 h; **(D)** Cell Counting Kit-8 (CCK-8) results of NP cells with TBHP (100 μM) and different concentrations of quercetin. **(E)** NP cells were treated with quercetin and TBHP after 24 h and imaged by phase-contrast microscopy (original magnification × 40, scale bar: 10 μm). **(F)** Migration was determined by scratch experiments. Cell migration distance = 0 h scratch width—24 h scratch width (original magnification × 40, scale bar: 10 μm). The data in the figures are represented as the mean ± *SD*. Significant differences between groups are indicated as ^∗∗^*P*< 0.01, ^∗^*P*< 0.05, *n* = 3. QUE, quercetin; TBHP, tert-butyl hydroperoxide.

Quercetin (3,3′,4′,5,7-pentahydroxyavone), a natural flavonoid (Figure 1A) present in many fruits and vegetables, showed many beneficial functions, including anticancer, anti-inflammatory, anti-aging and anti-oxidative properties in diverse degenerative diseases, and showed potential preventive and protective effects on osteoarthritis (Pallauf and Rimbach, 2013; Li et al., 2016; Feng et al., 2019; Hu et al., 2019). For example, quercetin has been confirmed to alleviate rat osteoarthritis by inhibiting inflammation and apoptosis of chondrocytes and modulating synovial macrophages polarization to M2 macrophages (Hu et al., 2019). Feng et al. (2019) reported that quercetin ameliorated the apoptosis of chondrocytes induced by oxidative stress via SIRT1/AMPK pathway. In addition, it has been demonstrated that quercetin could stimulate autophagy in various cell types including pulmonary arterial smooth muscle cells, human umbilical vein endothelial cells, macrophages and even tumor cells (Wang et al., 2011; He et al., 2017; Tomas-Hernandez et al., 2018; Cao et al., 2019; Rezabakhsh et al., 2019). However, whether quercetin has protective effect on IDD remain unclear.

Therefore, in this study, we used tert-butyl hydroperoxide (TBHP), a stable form of hydrogen peroxide, to trigger oxidative stress, which is widely accepted as an *in vitro* model to induce ECM degeneration and the apoptosis of NP cells. Then we investigated the protective effects of quercetin on apoptosis and ECM dyshomeostasis in nucleus pulposus cells under oxidative stress and also detected the activation of autophagy in a SIRT1-dependent manner under quercetin treatment. Finally, we evaluated the therapeutic effect of quercetin in a rat IDD model. Our study highlights the therapeutic potential of quercetin for IDD and confirms quercetin administration prevents IDD by activating SIRT1-autophagy in cellular and animal models.

## Materials and Methods

### Patient Samples

NP specimens were obtained from 12 patients (6 males and 6 females; mean age = 46.2 ± 19.6 years) with degenerative disc disease or scoliosis. The degree of IDD was assessed by 3 other blinded orthopedic researchers according to modified Pfirrmann grading system by magnetic resonance imaging (MRI). Grade II (*n* = 3) and III (*n* = 3) samples were combined into a moderate group (mean age = 40 ± 16.8), while Grade IV (*n* = 3) and V (*n* = 3) samples into a severe group (mean age = 52.3 ± 21.8). All the information of these samples was shown in Table 1. Ethics approval was obtained from the Institutional Review Board of Xijing Hospital of Fourth Military Medical University (KY20203146-1), and informed consent was obtained from each donor. The work presented in this paper was performed according to *The Code of Ethics of the World Medical Association* (Declaration of Helsinki).

**TABLE 1:** Demographic data of patients.

**Patient no.**	**Age**	**Gender**	**Level**	**Pfirrmann grading**
**Grade II/III**				
	22	F	L5/S1	II
	48	F	L5/S1	II
	25	M	L5/S1	II
	64	F	L4/L5	III
	30	M	L5/S1	III
	51	M	L4/L5	III
**Grade IV/V**				
	80	F	L4/L5	IV
	78	F	L4/L5	IV
	40	M	L5/S1	IV
	51	F	L5/S1	V
	32	M	L4/L5	V
	33	M	L5/S1	V

*F, female; M, male.*

### Reagents and Antibodies

Quercetin (purity ≥ 95%), TBHP and dimethylsulfoxide (DMSO) were purchased from Sigma-Aldrich (St Louis, MO, United States). 3-Methyladenine (3-MA) and Selisistat (EX-527) were purchased from Selleck Chemical (Houston, TX, United States). Cell Counting Kit-8 (CCK-8) was purchased from Dojindo (Kumamoto, Japan). Primary antibodies for MMP13 was purchased from Abcam (Cambridge, United Kingdom). Antibody for LC3, p62, cleaved-caspase3 and SIRT1 were purchased from Cell Signaling Technologies (Danvers, MA, United States) and antibody for Aggrecan was purchased from Millipore (Burlington, MA, United States). Horseradish peroxidase (HRP)-conjugated β-actin mouse monoclonal antibody, HRP-conjugated affinipure goat anti-mouse or goat anti-rabbit IgG (H + L), fluorescein (FITC)-conjugated affinipure goat anti-mouse or goat anti-rabbit IgG (H + L) and Cy3-conjugated affinipure goat anti-mouse or goat anti-rabbit IgG (H + L) were purchased from Proteintech (Wuhan, China).

### Cell Culture and Treatment

Rat NP cells were kindly generated and donated by Prof. Di Chen (Shenzhen Institutes of Advanced Technology, Chinese Academy of Sciences), and were maintained in DMEM/F12 (1:1) (DF12; Gibco, Grand Island, NY, United States) containing 10% fetal bovine serum (FBS; Invitrogen, Carlsbad, CA, United States) and 1% antibiotics (penicillin/streptomycin) (Gibco) in a 5% CO_2_ incubator at 37°C (Zheng et al., 2018; Xu X. et al., 2019). The complete culture medium was replaced every other day. The cells were used at passages 5–8. To establish an apoptosis and degenerative model of NP cells, complete culture medium with different concentrations (10, 25, 50, 75, 100, 125, 150, 200, and 400 μM) of TBHP were used for NP cells for 24 h. Cells were pretreated with different concentrations of quercetin (10, 20, 40, 60, 80, 120, 160, and 240 μM) for 2 h before the addition of TBHP (100 μM) to investigate its effect on cell apoptosis and degeneration. To study the role of autophagy and SIRT1 in quercetin-induced cell protection, NP cells were pretreated with 10 mM 3-MA (an autophagy inhibitor) or EX-527 (a SIRT1 enzymatic activity inhibitor) for 2 h before the addition of quercetin. All experiments were conducted with 3 replicates.

### Cell Viability Assay

Cell viability was evaluated by CCK8 according to the manufacturer’s instructions. Briefly, 5 × 10^3^ NP cells were seeded into 96-well plates with 3 replicates. After adhesion for 24 h, the cells were treated accordingly. The cells were washed with PBS, and then were incubated in a mixture of 10 μL CCK8 reagent and 100 μL fresh medium for 4 h at 37°C. Finally, the optical density of each well was measured by a microplate reader (BioTek, United States) at 450 nm.

### Transmission Electron Microscopy

After treatment for 24 h, NP cells were fixed in 2.5% glutaraldehyde overnight and then postfixed in 2% osmium tetroxide for 1 h. After dehydration in an ascending series of acetone, the samples were embedded into embedding medium (Epon 812) and cut into ultrathin sections by LKB-V ultramicrotome. Post-stained with uranyl acetate and lead citrate, the samples were visualized using a transmission electron microscope (TEM, H-7650; Hitachi, Tokyo, Japan).

### RNA Extraction and Quantitative Real-Time Polymerase Chain Reaction (qRT-PCR)

Total RNA was isolated from rat NP cells as treated above by using Total RNA Kit (Omega Biotek, Norcross, GA, United States) according to the manufacturer’s instructions. After isolation, virous groups of RNA were converted to cDNA using PrimeScript^TM^ RT Master Mix (TaKaRa, Tokyo, Japan). The synthesized cDNA was subjected to qRT-PCR using TB Green Premix Ex Taq II (TaKaRa, Tokyo, Japan). These reactions were performed in CFX96 (Bio-Rad, United States) and run pre-programmed program. The cycle threshold (Ct) values were collected and normalized to the level of respective GADPH. The △△Ct method was adopted as our previous study (Xu X. et al., 2019). The primers were listed as follows: Aggrecan (F) 5′-TGGCCTGCCTGACTTTAGTG-3′, (R) 5′-CCTGAACCACTGACGCTGAT-3′; MMP13 (F) 5′-GC AGCTCCAAAGGCTACAA-3′, (R) 5′-CATCATCTGGGAGC ATGAAA-3′; GADPH (F) 5′-CCACAGTCCATGCCATCAC-3′, (R) 5′-TCCACCACCCTGTTGCTGTA-3′.

### Western Blot Assay

NP cells were collected and lysed in RIPA buffer (Beyotime, Nantong, China) containing protease inhibitors (Beyotime). After centrifugation, the concentrations of protein were determined by BCA Protein Assay Kit (Beyotime). Each sample containing 30 μg protein was separated in sodium dodecyl sulfate-polyacrylamide gel (Beyotime) and transferred to nitrocellulose filter membranes (Millipore, Billerica, MA, United States). After blocking with 5% skim milk (skim-milk powder dissolved in Tris-buffered saline containing 0.1% Tween 20) for 1 h at 37°C, the membranes were incubated with primary antibodies against SIRT1 (1:1,000), p62 (1:1,000), LC3 (1:2,000), Aggrecan (1:1,000), MMP13 (1:1,000), and β-actin (1:2,000) at 4°C overnight. Then the membranes were incubated with the appropriate HRP-conjugated secondary antibodies (1:2,000) for 1 h at 37°C. Finally, the bands were detected with ECL-Plus Reagent (Millipore, Billerica, MA, United States) observed under Amersham Imager 600 (General Electric, United States).

### Immunofluorescence Staining

NP cells were fixed in 4% paraformaldehyde (Beyotime) for 15 min and permeabilized by 0.1% Triton X-100 (Beyotime) for 20 min. Then the cells were blocked by QuickBlock Blocking Buffer for Immunol Staining (Beyotime) for 1 h and incubated with primary antibody against LC3 (1:200), cleaved-caspase3 (1:350), Aggrecan (1:100), MMP13 (1:500), SIRT1 (1:100) at 4°C overnight. Then cells were incubated with appropriate FITC or Cy3-conjugated secondary antibodies (1:200) at 37°C for 2 h and finally incubated with 4′,6-diamidino-2-phenylindole (DAPI, Beyotime) for 2 min. Each step was followed by washing with PBS three times for 5 min. The cells or sections were observed under a fluorescence microscope and fluorescence signals were quantified with Image J software.

### Flow Cytometry Analysis

NP cells were seeded into a six-well plate at a density of 5 × 10^4^/mL and treated with different reagents for 24 h. After treatment, the NP cells were washed with PBS and trypsinized. The apoptosis of NP cells was assayed with Annexin V-FITC/PI kit (BD Biosciences, United States) according to the manufacturer’s instructions and the intracellular ROS was assayed with oxidation-sensitive fluorescent dye, 2′, 7′-dichlorofluorescein diacetate (DCFDA, Beyotime) by flow cytometry.

### Surgical Procedure

Eighteen male Sprague-Dawley rats (8-week-old) were randomly divided into three groups: Control group (*n* = 6), IDD group (*n* = 6), and QUE group (*n* = 6). All animal experiments were approved by the Animal Use and Care Committee of the Fourth Military Medical University and conducted in accordance with the National Institute for Health “Guide for the Care and Use of Laboratory Animals.” As previous studies described (Chen et al., 2016; Zhang et al., 2019), the rats in the IDD and QUE group were anesthetized with isoflurane and needles (27G) were used to fully puncture the whole layer of annulus fibrosus (Co7/8) through the tail skin, which was confirmed by a trial radiograph. The needle was kept in the disc for about 1 min and length of it was about 5 mm. After all the surgeries, these rats were subjected to intraperitoneal injection of 0.5 mL saline (Control group and IDD group) or quercetin (100 mg/kg, QUE group) three times a week for 8 weeks. Animal experimentation in this work met the *International Guiding Principles for Biomedical Research Involving Animals*.

### X-Ray and Radiological Analysis

After 8 weeks of needle puncture, each rat was randomly chosen to take X-ray before it was sacrificed using a cabinet X-ray imaging and irradiation systems (Faxitron Bioptic, LLC, Wheeling, IL, United States). All the images were analyzed by another experienced staff blinded to this study using a medical imaging software system (RadiAnt DICOM Viewer 4.6.9). Disc height index (DHI) was calculated as a previous study described (Han et al., 2008). Changes of the DHI were expressed as%DHI (%DHI = postoperative DHI/preoperative DHI × 100%).

### Histopathologic Analysis

The spine specimens of rats were fixed in 4% paraformaldehyde for 48 h, decalcified in 10% ethylenediaminetetraacetic acid (EDTA; pH 7.4) for 30 days and embedded into OCT. Midsagittal-oriented sections (5 μm) were prepared for histological staining. Hematoxylin-eosin (HE) and safranin O-fast green (SO) staining were performed by using respective staining kit according to standard protocols (Solarbio, Beijing, China). Histological scores were evaluated as described previously (Han et al., 2008).

### Statistical Analysis

The results were given as means ± SD. Statistical analyses were performed using SPSS 22.0 and GraphPad Prism 7.0 software. Differences between two groups were analyzed by Student’s *t*-test, while differences among multiple groups were analyzed by one-way analysis of variance followed by Tukey’s multiple-comparison *post hoc* test. Statistical significance was set at *P* < 0.05.

## Results

### The Effects of Quercetin and TBHP on the Viability of NP Cells

To evaluate the effect of quercetin and TBHP on the viability of NP cells, cells were treated with various concentrations quercetin and TBHP for 24 h, and the viability was examined by CCK8 assay. As shown in Figure 1B, quercetin showed no significantly cytotoxic effect to NP cells after 24 h treatment at concentrations up to 160 μM. For TBHP treatment, the NP cells showed decreased viability in a dose-dependent manner (Figure 1C) and 100μM TBHP was accepted for *in vitro* model. Quercetin showed evident protective effect against the TBHP-induced cell death and 60 μM quercetin showed the optimal therapeutic effect (Figure 1D).

After 100 μM TBHP treatment for 24 h, NP cells shrank in size, more floated cells were observed and less attached cells remained, while quercetin significantly reversed this phenomenon (Figure 1E). Moreover, 100 μM TBHP notably inhibited the migration of NP cells and quercetin also recovered the migration capacity (Figure 1F).

### Quercetin Induced Autophagy and SIRT1 Expression in the NP Cells

**FIGURE 2:**
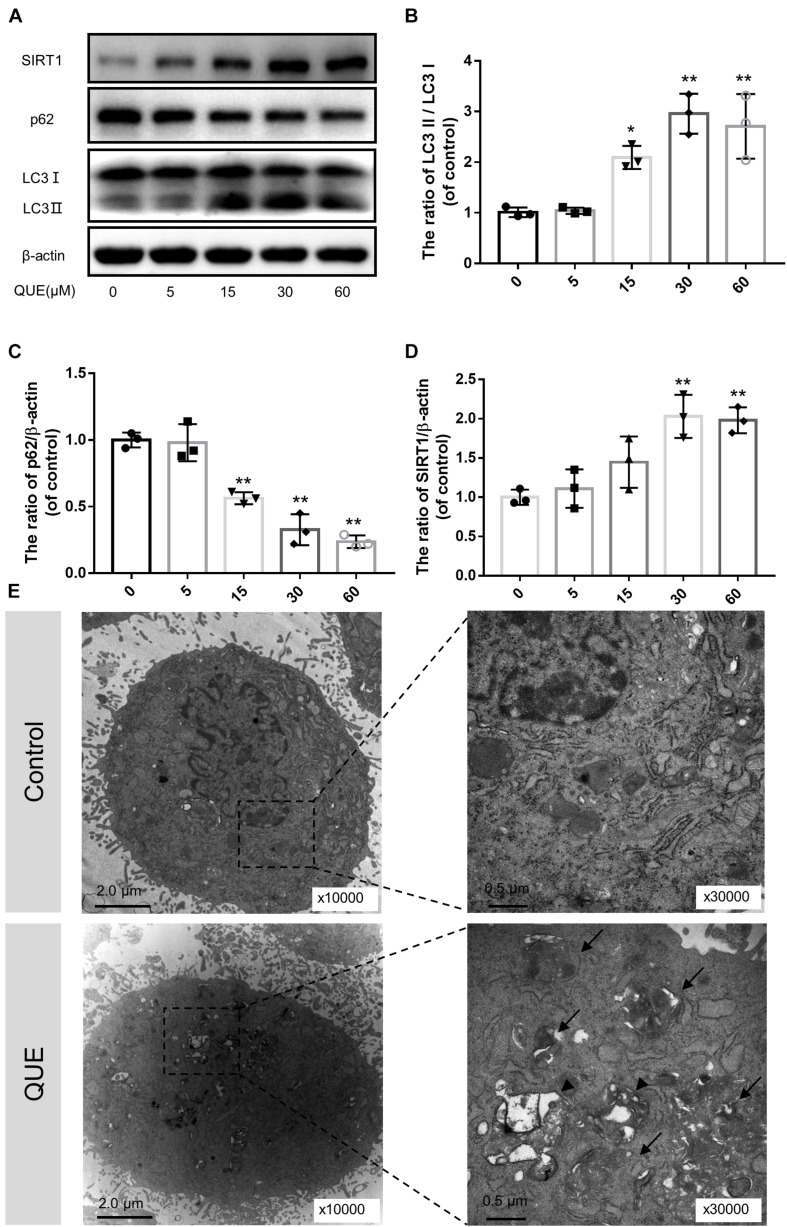
Quercetin treatment induces autophagy and activates SIRT1 in the NP cells. **(A–D)** The NP cells were incubated with 0, 5, 15, 60, or 100 μM quercetin for 24 h. Protein content of SIRT1, LC3 and p62 were detected by western blot. **(E)** Autophagosomes and autophagolysosomes were detected by transmission electron microscopy (Black arrow, autophagosome; black triangle, autophagolysosome). The data in the figures represent the mean ± *SD*. ^∗∗^*P* < 0.01, ^∗^*P* < 0.05, *n* = 3.

To investigate the effect of quercetin on autophagy in the NP cells, we examined the expression of LC3 and p62/SQSTM1 by western blot. As shown in Figures 2A–D, the ratio of LC3-II/LC3-I was increased in a dose-dependent manner 24 h after quercetin treatment. While the level of p62 decreased in a dose-dependent manner as well. Interestingly, the protein level of SIRT1 increased in a dose-dependent manner as well, which was consistent with the activation of autophagy.

Moreover, we observed autophagosomes and autophagolysosomes, two key structures in autophagy, by transmission electron microscopy (TEM). Compared with the control group, the quercetin-treated NP cells showed more autophagosomes and autophagolysosomes in the cytoplasm, which confirmed the activation of autophagy (Figure 2E).

### Quercetin Ameliorated Apoptosis and Reduced ROS Level of NP Cells via the Activation of Autophagy

**FIGURE 3:**
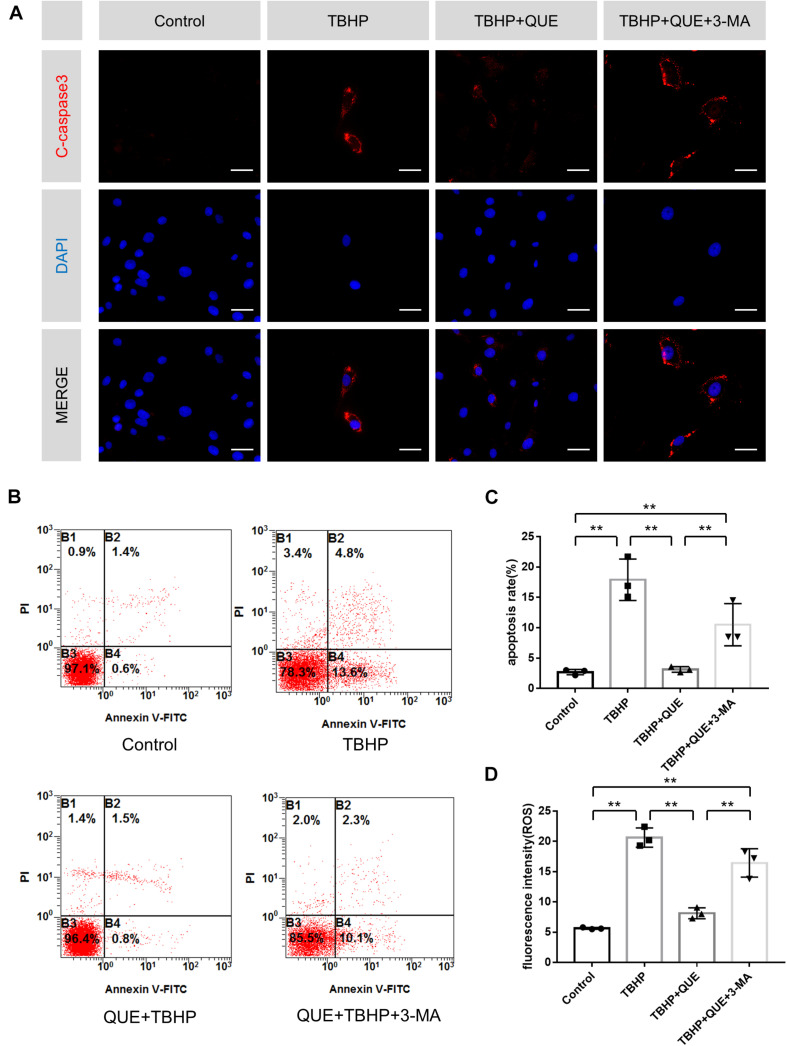
Quercetin inhibits apoptosis and reduces ROS level of NP cells via the activation of autophagy. **(A)** Immunofluorescence of cleaved-caspase 3 protein in the NP cells (scale bar: 25 μm). **(B,C)** Flow cytometry was used to detect the apoptosis of the NP cells. **(D)** Relative levels of reactive oxygen species (ROS) were evaluated by DCFH-DA. The data in the figures represent the mean ± *SD*. ^∗∗^*P* < 0.01, *n* = 3.

To investigate whether quercetin protected NP cells against apoptosis via autophagy, autophagy inhibitor 3-MA was used. Immunofluorescence of cleaved-caspase3 showed that quercetin significantly reduced the TBHP-induced expression of cleaved-caspase3 and 3-MA reversed this effect of quercetin (Figure 3A). Moreover, the flow cytometry results also confirmed that 3-MA inhibited the anti-apoptosis effect of quercetin (Figures 3B,C).

ROS-triggered oxidative stress can induce mitochondrial dysfunction in NP cells, which constitutes one major reason of apoptosis. Hence DCFDA staining was used to evaluate the ROS level in NP cells. Compared with the TBHP group, quercetin treatment significantly reduced the ROS level and this effect was markedly block by 3-MA (Figure 3D).

### Quercetin Inhibited TBHP-Induced ECM Degeneration via Autophagy

**FIGURE 4:**
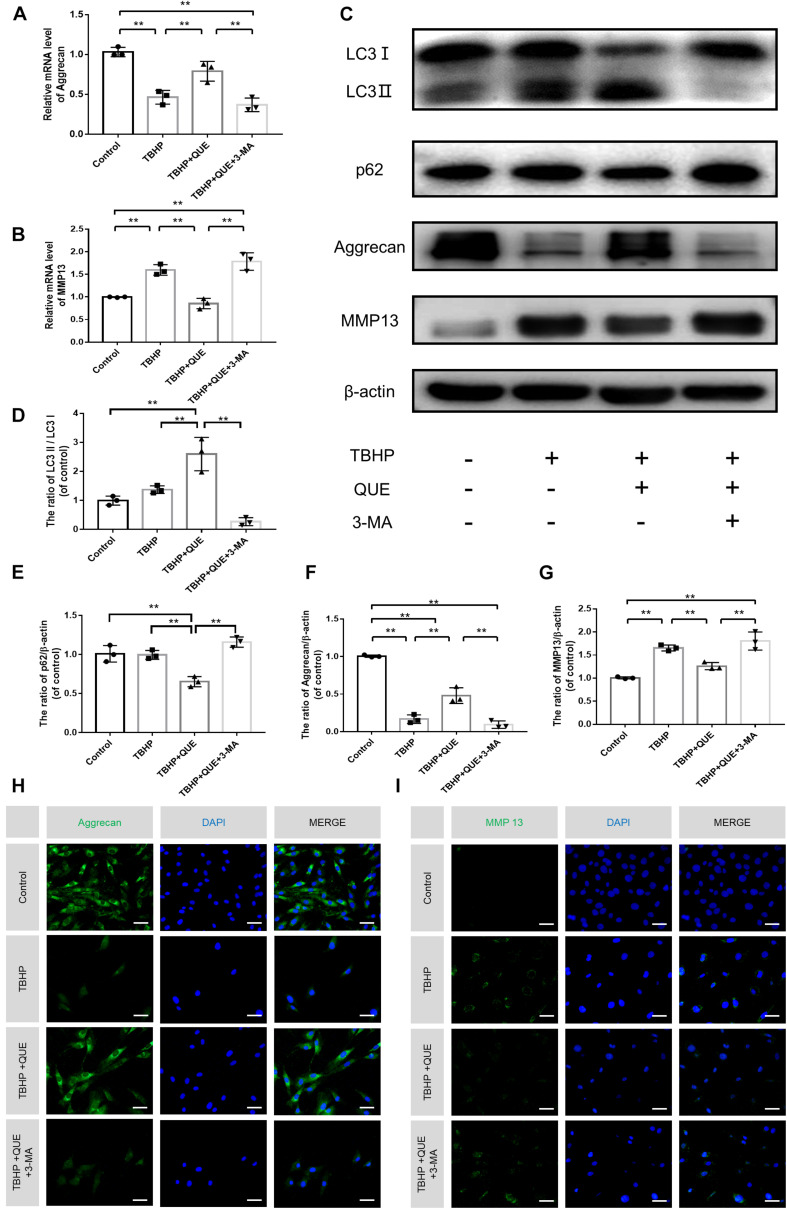
Quercetin ameliorates the degeneration of the NP cells via autophagy. **(A,B)** The mRNA levels of Aggrecan and MMP13 were measured by qRT-PCR. **(C–G)** The protein expression of Aggrecan, MMP13, LC3, and p62 in NP cells treated above. **(H,I)** Immunofluorescence of Aggrecan and MMP13 in the NP cells (scale bar: 25 μm). The data in the figures represent the mean ± *SD*. ^∗∗^*P*< 0.01, *n* = 3.

To evaluate the degree of degeneration, major ECM synthesis gene *Acan* and ECM degrading gene *Mmp13* were detected by qRT-PCR. As shown in Figures 4A,B, TBHP significantly reduced the mRNA level of *Acan* and increased the mRNA level of *Mmp13*. Quercetin successfully reversed this phenotype by activation of autophagy, which was confirmed by autophagy inhibitor 3-MA. The western blot and the immunofluorescence results showed that the protein expression of Aggrecan and MMP13 was consistent with qRT-PCR results (Figures 4C,F–I). Compared with the control group and TBHP group, 60 μM quercetin significantly activated autophagy in NP cells and 3-MA reversed the increased ratio of LC3-II/LC3-I and decreased level of p62 (Figures 4C–E and Supplementary Figures 1A,B).

### Quercetin Protected NP Cells Against Apoptosis and Prevented ECM Degeneration via SIRT1-Mediated Autophagy Stimulation

**FIGURE 5:**
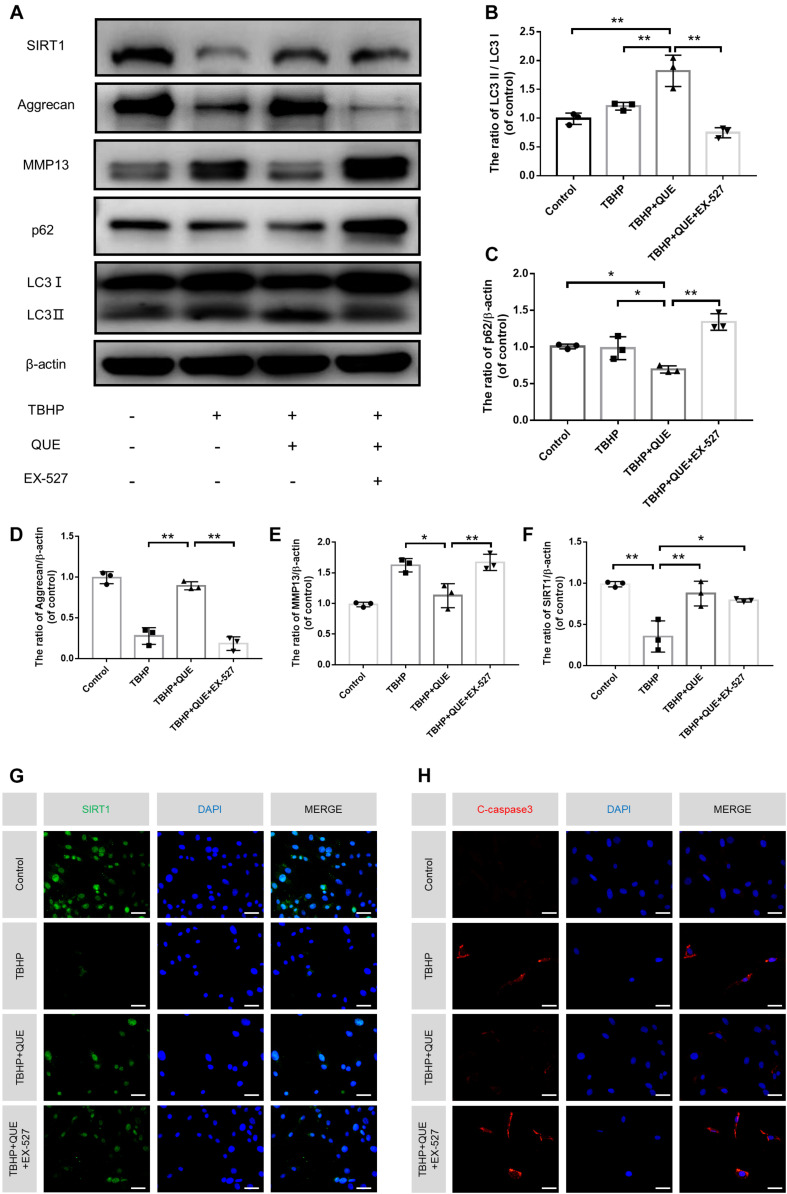
Quercetin ameliorates the apoptosis and degeneration of the NP cells via SIRT-autophagy pathway. NP cells were treated with culture medium (DF12 + 10% FBS, Control group), or TBHP alone (100 μM, TBHP group), or quercetin (60 μM) and TBHP (TBHP + QUE group), or TBHP, quercetin and EX-527 (10 μM, TBHP + QUE + EX527 group). **(A–F)** The protein levels of SIRT1, Aggrecan, MMP13, p62, and LC3 in the NP cells were detected by western blot. **(G,H)** The protein expression of Aggrecan, MMP13 in NP cells treated above. **(F,G)** The expression of SIRT1 and cleaved-caspase3 in the NP cells were assessed by immunofluorescence analysis (scale bar: 25 μm). The data in the figures represent the mean ± SD. ^∗∗^*P* < 0.01, ^∗^*P* < 0.05, *n* = 3.

Previous studies reported that quercetin could promote the expression of SIRT1, which was an upstream regulator of autophagy. In order to investigate whether quercetin inhibits TBHP-induced apoptosis and degeneration, and activates autophagy via the activation of autophagy, specific SIRT1 enzymatic activity inhibitor EX-527 was used. The western blot results showed that quercetin treatment partially recovered TBHP-induced the decrease of SIRT1, activated autophagy (increased ratio of LC3-II/LC3-I and decrease P62 level) and rescued degenerative phenotypes (increased expression of Aggrecan and decreased expression of MMP13). However, EX-527 abolished the protective effects of quercetin and inhibited quercetin-mediated autophagy (Figures 5A–F). The immunofluorescence results of cleaved-caspase3 and SIRT1 also confirmed that quercetin protected NP cells against apoptosis via SIRT1 activation (Figures 5G,H).

### Decreased SIRT1 Expression and Autophagy Stimulation in Human Degenerated IVDs

**FIGURE 6:**
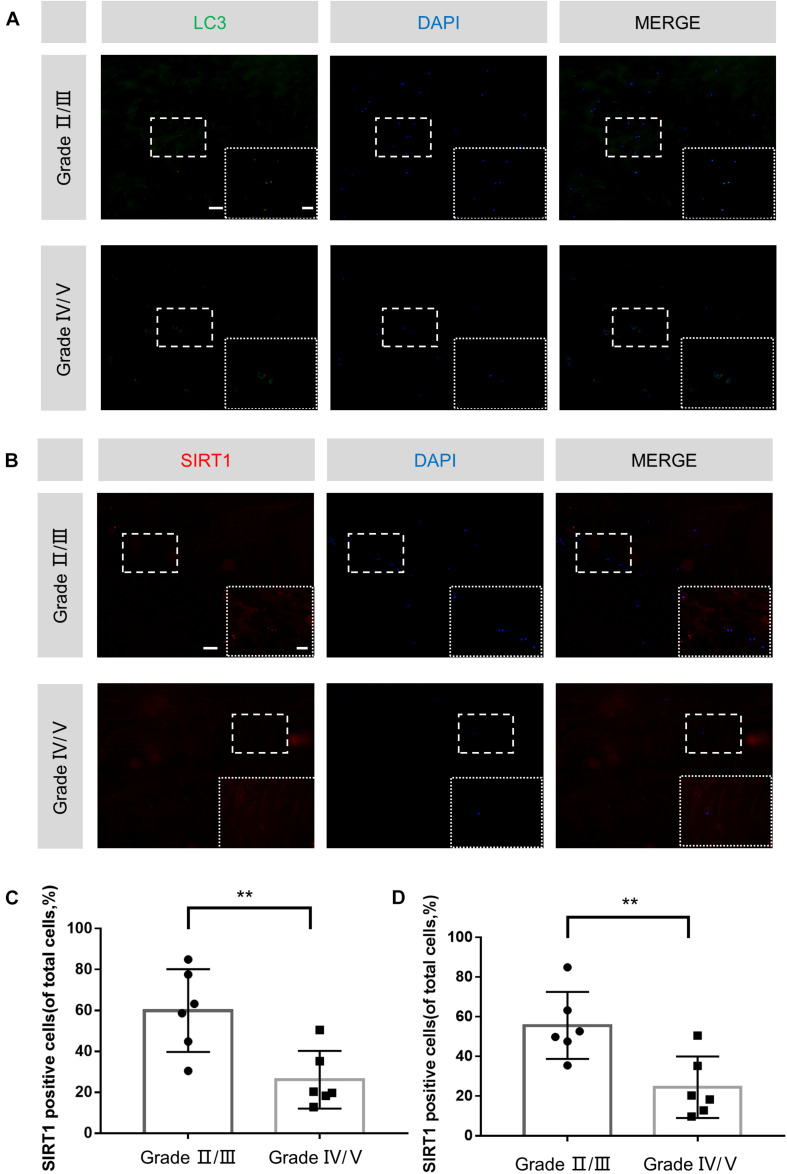
Decreased SIRT1 expression and autophagy stimulation in human degenerated IVDs. **(A–D)** Immunofluorescence staining and quantitative analysis of LC3 and SIRT1 in human NP samples (original magnification × 100, scale bar: 100 μm, original magnification × 400, scale bar: 25 μm). The data in the figures represent the mean ± *SD*. ^∗∗^*P*< 0.01, *n* = 6.

Previous studies showed that SIRT1 and autophagy were closely associated with degenerative diseases. Therefore, we examined the expression level of SIRT1 and LC3 in human NP tissues with different degeneration grades by immunofluorescence. A total of 12 degenerative IVDs were taken into investigation and concrete details were shown in Table 1. Compared with Grade IV/V group, higher proportion of LC3-positive cells and SIRT1-positive cells were observed in Grade II/III group (Figures 6A–D). Therefore, we concluded that SIRT1 downregulation and inhibition of autophagy were correlated with IDD progression.

### Quercetin Treatment Ameliorated Rat IDD in a Needle Puncture Model

**FIGURE 7:**
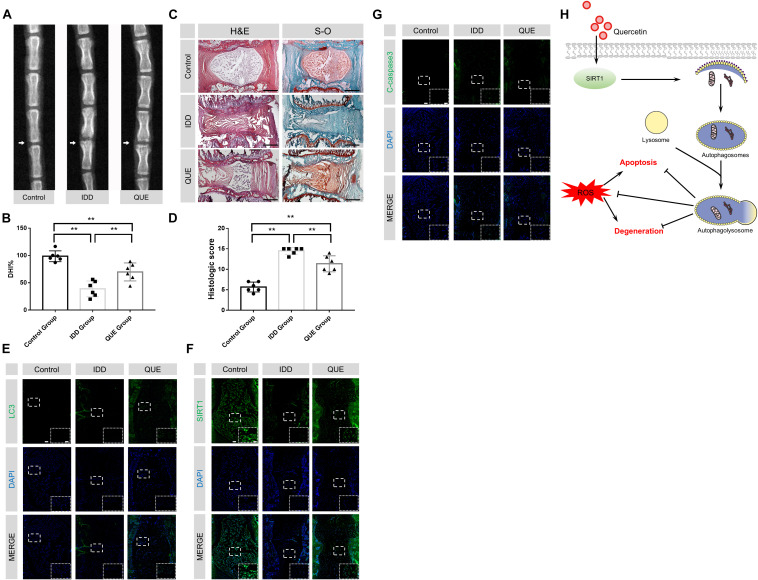
Quercetin treatment ameliorated rat IDD *in vivo*. **(A,B)** Representative X-ray images of rat coccygeal vertebrae on days 56 after puncture and the percent disc height index (DHI%). **(C,D)** Representative HE and SO staining images and corresponding histological scores (original magnification × 40, scale bar: 500 μm). **(E–G)** Immunofluorescence staining and quantitative analysis of LC3, SIRT1 and cleaved-caspase3 in intervertebral NP tissues of the rat (original magnification × 40, scale bar: 100 μm, original magnification × 200, scale bar: 20 μm). **(H)** Proposed protective mechanism of quercetin on IDD. As one of the main inducers for IDD, ROS may lead to apoptosis and degeneration in NP cells. Quercetin may promote the expression and activity of SIRT1, and then activate autophagy. Activated autophagy induced by quercetin may downregulate the ROS level in NP cells and inhibit oxidative stress-induced apoptosis and degeneration. The data in the figures represent the mean ± *SD*. ^∗∗^*P*< 0.01, *n* = 6.

To evaluate the therapeutic effects of quercetin *in vivo*, a classic disc degeneration model induced by needle puncture was adopted. The X-ray results at 56 d showed significant reduction of disc height when compared with the control group. This narrowing process was partially inhibited after quercetin treatment (Figures 7A,B). Moreover, HE and SO staining were performed to evaluate histological structure of NP tissues. As shown in Figures 7C,D, the number of NP cells significant decreased and the less ECM was preserved 56 d after needle puncture. Compared with the Control group, the histological score of the IDD group was much higher. Despite some problems, such as decreased size of the NP and inward bulging of the inner annulus still existed, quercetin administration significantly alleviated IDD phenotype induced by needle puncture. No surprisingly, the histological score of the QUE group was also lower than that of the IDD group. Compared with the IDD group, more LC3-positive cells and SIRT1-positive cells and less cleaved-caspase3-positive cells were observed in the QUE group, which confirmed that quercetin protected NP cells against apoptosis and prevented ECM degeneration via the SIRT1-autophagy pathway (Figures 7E–G).

## Discussion

Intervertebral disc degeneration is one of the major causes of LBP, which showed very negative impacts on people’s quality of life (Sun et al., 2013; Molladavoodi et al., 2019; Taylor and Bishop, 2019). The frequently-used treatments at present for patients with IDD are non-steroidal anti-inflammatory drugs (NSAIDs) or just some relaxation to relieve some symptom. However, these treatments showed no help for preventing or retarding IDD (Madigan et al., 2009). Due to the substantial morbidity and limited treatment modalities, it is necessary for us to search for new approaches to delay or even reverse the progress of IDD. Our studies demonstrated for the first time that quercetin was able to activate SIRT1-dependent autophagy to inhibit TBHP-induced apoptosis of NP cells and ECM degeneration. *In vivo*, quercetin could also alleviate the progression of IDD induced by needle puncture via the SIRT1-autophagy pathway.

Previous studies have demonstrated that oxidation and oxidation products are positively associated with IDD. As one of the key pathogenic mechanisms underlying IDD, altered ECM metabolism of NP cells could result in structural failure of ECM and then decreases the tolerance of disc to mechanical loading. Decreased synthesis and increased degradation of ECM putatively characterize the disrupted ECM metabolism, and more importantly, the causative role of over-produced ROS has been becoming a well-entrenched dogma suggested by our study and many others (Feng et al., 2017; Li et al., 2018; Zhang et al., 2020). On the one hand, numerous studies have shown that H_2_O_2_ significantly downregulated the expression of collagen type II and aggrecan in human and rat disc cells. ROS overproduction induced by proinflammatory cytokines or high oxygen tension prominently suppressed the matrix synthesis and upregulated the expression of matrix degradation proteases in human and rat disc cells (Yang et al., 2014; Dimozi et al., 2015; Chen et al., 2016). On the other hand, oxidized collagens are more vulnerable to matrix proteases (Cannizzo et al., 2012; Scharf et al., 2013). Therefore, we examined ECM metabolism imbalance under oxidative stress.

Autophagy is an evolutionarily conserved regulatory behavior for the maintenance of cellular homeostasis via lysosome-dependent degradation of damaged organelles and misfolded proteins (Mizushima and Komatsu, 2011; Wang et al., 2019). Several studies indicated that autophagy was able to protect IVD cells against apoptosis, senescence and degeneration. Chen et al. reported that metformin protected NP cells against apoptosis and senescence via activation of AMPK-dependent autophagy (Chen et al., 2016). Chen et al. reported that melatonin, an endogenous hormone synthesized by the pineal gland and many other organs, could also exert a protective effect on NP via activating autophagy (Chen et al., 2020). However, due to limited raw material sources and various side effects of traditional drugs, it is quite necessary to screen a new drug to ameliorate IDD via autophagy stimulation.

Quercetin is a member of the flavonoid family, which ubiquitously exists in fruit, vegetables, tea and many other edible plants, indicating a low cost and broad source characteristic. The isolation and biological recognition of it could date back to 1936 (Patel et al., 2018). Previous studies proved that quercetin had protective effect on many degenerative diseases via activating autophagy and promoting the expression of SIRT1 (Chung et al., 2010; Costa et al., 2016). Recent years, abundant clinical trials of quercetin in various diseases has been implemented, which showed protective effects on hypertension, hyperlipemia, chronic prostatitis syndromes and many other chronic diseases (Shoskes et al., 1999; Mulholland et al., 2001; Edwards et al., 2007; Egert et al., 2009). Our study for the first time demonstrated that quercetin could activate autophagy in a SIRT1-dependent pathway in NP cells.

It is generally accepted that apoptosis of NP cells and degeneration of ECM are two main causes of IDD (Chen et al., 2016; Xu X. et al., 2019). To investigate whether quercetin inhibited the apoptosis of NP cells and ECM degeneration via autophagy, classical autophagy inhibitor 3-MA was used in this study. After 3-MA treatment, the quercetin-induced autophagy was inhibited, which was confirmed by decreased protein level of p62 and increased ratio of LC3-II/LC3-I. At the same time, the protective effect of quercetin for IDD was also abolished. In this study, we found that the expression of cleaved-caspase3, a critical apoptosis indicator, was increased with the combination of quercetin and 3-MA under oxidative stress. Flow cytometry analysis also confirmed that combined treatment with 3-MA and quercetin increased apoptosis rate and ROS level compared to quercetin treatment alone under TBHP-induced oxidative stress. Moreover, quercetin promoted the anabolism of ECM (increased aggrecan) and inhibited the catabolic process (decreased MMP13), which was also reversed with the 3-MA treatment.

To reveal the relationship among SIRT1, autophagy and IDD, EX-527, a classical SIRT1 enzymatic activity inhibitor was applied in this study. Although EX-527 did not change the expression of SIRT1, it is efficient to inhibit the enzymatic activity of SIRT1, which then inhibit the activation of autophagy and recovery of degeneration induced by quercetin.

In order to further confirm the protective effect of quercetin on IDD via the SIRT1-autophagy pathway, Immunofluorescence staining of human NP tissues with different pfirrmann grading and *in vivo* experimentation on rats were implemented. Our data is the first to indicate that both the percentage of LC3 and SIRT1 positive cells decreased in the NP tissues of severe IDD group, which suggested negative correlation between SIRT1 expression as well as autophagic level and the degree of disc degeneration. *In vivo* data also confirmed that quercetin successfully ameliorated needle puncture-induced IDD via the increased level of SIRT1 and activation of autophagy.

Interestingly, our study seems to contradict some recent studies. Ye et al. (2013) reported that macroautophagy was present and associated with accelerated pathological process of IVDD in rats. However, their animal model was quite different with our work and the degenerative degree was considerably moderate. Gene expression data of their work came from AF tissue, which is also different from ours. In addition, Gruber et al. (2015) showed an upregulation of autophagy in more degenerated human AF tissue. A totally different grading standard (we used modified Pfirrmann grading system rather than Thompson system) might also contribute to the discrepancy between the studies. Moreover, Jiang et al. suggested that NP cells from degenerative discs exhibited less autophagosomes number and lower expression of LC3 and Beclin-1 (Jiang et al., 2014). While Quan et al. (2020) showed that LC3-II expression in grade III was significantly higher than in grade II, IV, and V. These results suggested that autophagy activation might occur at the early stage of IDD and play a protective role. As a result, the autophagy activation in moderate group might indicate a potential therapeutic target for IDD. In addition, excessive autophagy could also contribute to cell death (type II programmed cell death), which might promote the progression of IDD. Zhan et al. (2019) reported that overexpression of HOTAIR (a long non-coding RNA) could enhance autophagy and lead to IDD phenotype. Our data did not fully contradict to this conclusion. In addition, in our *in vivo* experiment, 8 weeks needle puncture model resulted in a severe degeneration of NP tissue, which was confirmed by HE and SO staining. Immunofluorescence staining of LC3 showed less LC3-positive cells in IDD group, which supported our speculation. Our CCK8 assay showed that over 80 μM quercetin treatment had no protective effects on TBHP-induced decrease of cell viability, although quercetin could activate autophagy in a dose-dependent manner. Thus, moderate rather than excessive autophagy induced by quercetin (60 μM) is an effective protecting strategy.

In addition, except for the regulatory effects on SIRT1, quercetin also showed anti-aging or anti-inflammatory effects by activating Keap1/Nrf2 pathway (Liu et al., 2015; Shao et al., 2019; Xu M. X. et al., 2019). Although our data from western blot or immunofluorescence staining strongly suggested that quercetin activated autophagy and increased SIRT1 expression, we could not exclude other regulatory effects. We would pay more attention on other mechanism of quercetin on IDD in future research.

In conclusion, our study provides the evidence that quercetin treatment induces autophagy in a SIRT1-dependent manner to protect NP cells against apoptosis and prevent ECM degeneration induced by oxidative stress (Figure 7H). Autophagy specific inhibitor 3-MA and SIRT1 specific enzymatic activity inhibitor EX-527 unsurprisingly abolish these protective effects. These results suggest that quercetin treatment can be considered a potential therapeutic method for the prevention of IDD.

## Data Availability Statement

The raw data supporting the conclusions of this article will be made available by the authors, without undue reservation, to any qualified researcher.

## Ethics Statement

The studies involving human participants were reviewed and approved by the Institutional Review Board of Xijing Hospital of Fourth Military Medical University. The patients/participants provided their written informed consent to participate in this study. The animal study was reviewed and approved by Animal Use and Care Committee of the Fourth Military Medical University.

## Author Contributions

LY and DoW designed the experiments. DoW, XH, and DiW carried out most of the experiments. PP, XX, BG, CZ, HW, and ZS helped to collect the samples. HJ and QS proofread the manuscript. DoW, LY, and ZL supervised the experiments, analyzed the results, and wrote the manuscript. All authors contributed to the article and approved the submitted version.

## Conflict of Interest

The authors declare that the research was conducted in the absence of any commercial or financial relationships that could be construed as a potential conflict of interest.
