# Supplementary material for: Quercetin Suppresses Apoptosis and Attenuates Intervertebral Disc Degeneration via the SIRT1-Autophagy Pathway
Source: Front Cell Dev Biol. 2020 Dec 10;8:613006. doi: 10.3389/fcell.2020.613006 (PMC7758489; doi:10.3389/fcell.2020.613006)
Supplement: Supplementary file 1 [file Data_Sheet_1.DOCX]

Supplementary Material
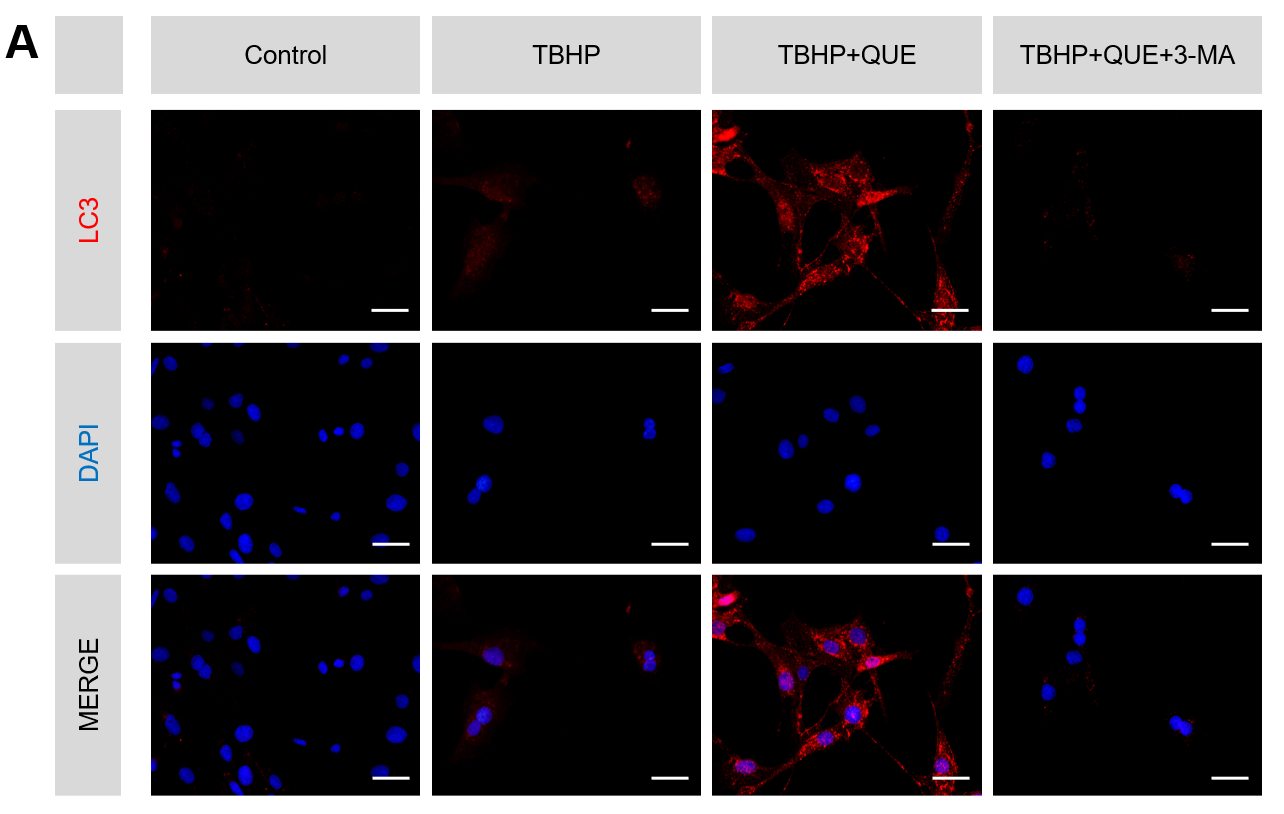

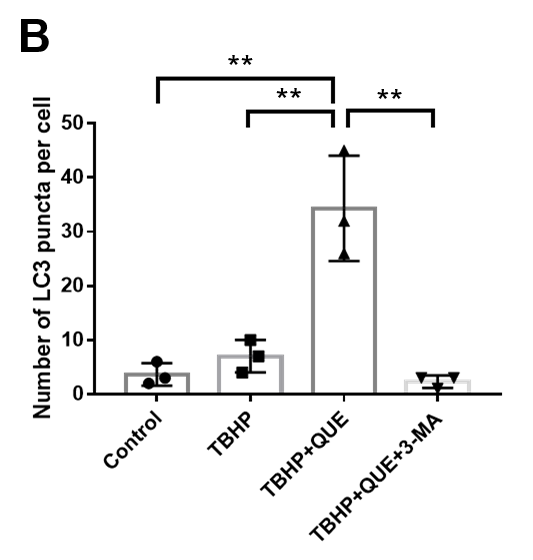


**Supplementary Figure 1.** Figure 3. 3-MA inhibited the quercetin-induced autophagy in the NP cells. NP cells were treated with culture medium (DF12+10%FBS, Control group), or TBHP alone (100μM, TBHP group), or quercetin (60 μM) and TBHP (TBHP+QUE group), or TBHP, quercetin and 3-MA (10 mM, TBHP+QUE+3-MA group). (A). Immunofluorescence of LC3 protein in the NP cells (scale bar: 25 μm). (B). Quantitative analysis of LC3 puncta per cell. The data in the figures represent the mean ± S.D. **P＜0.01, *P＜0.05, n=3.
